# Updating Our Knowledge on Fish Immunology

**DOI:** 10.3390/ijms241310856

**Published:** 2023-06-29

**Authors:** Elena Chaves-Pozo, Alberto Cuesta

**Affiliations:** 1 Centro Oceanográfico de Murcia, Instituto Español de Oceanografía (COMU-IEO), CSIC, Carretera de la Azohía s/n, Puerto de Mazarrón, 30860 Murcia, Spain; 2 Immunobiology for Aquaculture Group, Department of Cell Biology and Histology, Faculty of Biology, University of Murcia, 30100 Murcia, Spain

Fish is the first group of vertebrates to appear during animal evolution. It is the largest and most diverse vertebrate group, with more than 34,000 species inhabiting most of the world’s aquatic environments. In fact, it is considered a paraphyletic group distinct from tetrapods, the rest of the vertebrates. Beside our basic knowledge of their biological, evolutionary, and environmental aspects, fish have long been established as a relevant source for human diet, mainly due to their abundance of essential omega-3 fatty acids and certain minerals. To cover this demand, fisheries and aquaculture are the techniques employed to catch or culture them, respectively, whereby aquaculture has now surpassed fisheries in terms of production and represents an industry with very high productivity in biomass and economic terms. Therefore, our knowledge of the biology of cultured species is vital. One of the principal aspects is the characterization of the immune response of this animal group, in which appeared, from an evolutionary point of view, the first complete immune response with both innate and acquired immunity. Thus, the main objective of this collection is to provide new insights into fish immunity, which is mandatory to improve fish health and welfare but also control fish diseases and economical losses.

The published articles under this collection will be grouped as follows:

1. Deepen the identification and characterization of immune cells and molecules of the fish immune system

Classical studies conducted to identify the structure, localization, and distribution of immune cells are still mandatory due to the diversity of species and immune organs. This knowledge is the starting point to design treatments as immunostimulants or vaccines for preventing and controlling diseases. Thus, Lauriano et al. [1] performed a classical immunohistochemistry (IHC) study of the gut-associated lymphoid tissue (GALT) in the African bonytongue (*Heterotis niloticus*, Cuvier 1829). They used some antibodies and identified different types of leukocytes, including dendritic-like cells and CD4+ve lymphoid cells, in the intestinal submucosae. The cells were grouped in ovoid structures resembling those of the mammalian Peyer patches, an observation that is uncommon in fish and deserves further evolutionary studies. On the other hand, Liu et al. [2] evaluated the Rag1 and Rag2 gene expression in the rice-field eel (*Monopterus albus*) by real-time PCR (qPCR) and in situ hybridization (ISH). They found that lymphocytes start to be produced at 2 days post-hatching in the thymus, being the main and first lymphopoietic tissue, and mature later in liver and head-kidney.

Another group of studies identify an immune candidate gene by conducting an in silico analysis followed by its tissue distribution and regulation in vitro and in vivo, usually upon bacterial infection. These studies contribute to deepen and widen our knowledge of the immune response of fish. Included in this Special Issue, these studies dealt with highly relevant fish species for the aquaculture sector and local human supply in Asiatic countries as well as worldwide, as in the case of tilapia. Therefore, the following studies focused on the inflammasome component apoptosis-associated speck-like protein containing a CARD domain (ASC) in the large yellow croaker (*Larimichthys crocea*) [3]; the B-cell adaptor for phosphoinositide 3-kinase (BCAP) homolog in the lamprey (*Lampetra japonica*) [4]; the CD68 gene from the Wuchang bream (*Megalobrama amblycephala*) (Cui et al.); the complement factor I (CFI) gene in the grass carp (*Ctenopharyngodon Idella*) [5]; and the complement C3 protein [6], the serum amyloid P component (SAP) [7], the vasoactive intestinal peptide (VIP) and its receptor (VIPR1) [8], and the tartrate-resistant acid phosphatase type 5b (TRAP5b) [9] genes in the Nile tilapia (*Oreochromis niloticus*). Interestingly, Nile tilapia specimens with over or knockdown expression of TRAP5b showed significantly lower or higher bacterial loads, respectively, upon in vivo infection with *Streptococcus agalactiae* [9]. In addition to expression data, some of the studies produced the recombinant protein (r) and evaluated its biological activity: rC3 protein reduced the *S. agalactiae*-induced inflammation and tissue damage in vivo while promoting the phagocytosis by monocyte/macrophages [6]; rCD68 showed agglutination of *Escherichia coli* but not *Aeromonas hydrophila* [10]; rSAP binds to *S. agalactiae* or *A. hydrophila* and shows an opsonizing capacity for macrophages as well as a complement-mediated lysis of erythrocytes [7]; rCFI protein reduced the content of C3b in the serum [5]; and rTRAP5b increased the immune response of head-kidney macrophages by priming the phagocytic activity, and it increased the production of reactive oxygen species (ROS) or inflammatory cytokines [9]. It is very interesting to note how CD68 antiserum was generated to help identify CD68+ve cells and how they increased upon bacterial challenge and by LPS incubation, suggesting that it could be considered a good marker for macrophages [10]. In addition, lamprey BCAP protein levels, and its tyrosine kinase activity, were increased in peripheral blood lymphocytes, gills, and supraneural myeloid bodies upon bacterial infection as well as via lipopolysaccharide (LPS) stimulation, but not phytohemagglutinin (PHA), thus pointing to a role in the B-cell/LPS-mediated immune response [4].

2. Omics for depicting fish immunity

In contrast to the characterization of one concrete molecule, as described above, the use of omics technologies helps to identify the pathways and families of molecules relevant in a process. However, omics studies in non-model fish species are difficult due to the lack of proper databases, incomplete annotations, and the great variability in most of the relevant immune genes, all of which characterizes fish diversity. In this regard, here, we show the compilation of four transcriptomic studies and one metabolomic study. Maekawa et al. [11] performed a transcriptomic analysis in the head-kidney and spleen of East Asian fourfinger threadfin fish (*Eleutheronema tetradactylum*) after infection with a strain of *S. iniae* isolated from a farm outbreak. Genes up-regulated, and probably involved in the fish immunity against bacteria, were found to relate to phagosome, Th1, and Th2 cell differentiation, complement and coagulation cascades, hematopoietic cell lineage, antigen processing and presentation, and cytokine–cytokine receptor interaction pathways, thus highlighting the role of inflammatory cytokines (IL-1β, IL-6, IL-11, IL-12, IL-35, and TNF) and chemokines (CXCL8 and CXCL13). Ning et al. [12] performed a transcriptomic and metabolomic study in the yellow catfish (*Pelteobagrus fulvidraco*) against *A. veronii*. During infection, fish tended to increase the synthesis of lipids and to induce the inflammatory response, ROS production, and cell apoptosis while reducing the mitotic potential. After infection, during the recovery period, processes such as sugar catabolism, energy generation, antioxidant protection, cell proliferation, and autophagy were promoted. Zhao et al. [13] evaluated the benefits of dietary mannan oligosaccharides (MOS), a well-known immunostimulant, on the intestinal immune response of the Wuchang bream against *A. hydrophila* infection. They found that MOS improved pathways relating to complement and coagulation cascades, natural killer cell-mediated cytotoxicity, Fc gamma R-mediated phagocytosis, and antigen processing and presentation in the midgut, while the stability of the intestinal barrier seemed to be maintained. Unfortunately, protection or any other functional evaluation to link pathways/processes with resistance or phenotypes was not provided. Strikingly, one of the studies evaluated the impact of different temperatures during the larval rearing of European sea bass (*Dicentrarchus labrax*) at phenotypic and transcriptomic levels, specifically, the miRNAs through sncRNA high-throughput sequencing [14]. They found that temperature influences the abundance of miRNA, mainly those associated with sex determination and immune response, in particular, those relating to B-cell activation. Therefore, future omics studies in fish should consider including other important determinations to link the molecules (usually mRNA) with functions.

3. Immunity to pathogens

In contrast to the studies presented above, which rely on fish immunity upon infection, the study performed by García-Álvarez et al. [15] also focuses on the pathogen. Thus, the study demonstrates that resistance and immunity of gilthead seabream (*Sparus aurata*) larvae against natural reassortant nodavirus (NNV) depend on age and reassortant strain. Interestingly, the differences observed in resistance were associated with antimicrobial peptides (AMP), at either gene or protein levels, cell-mediated cytotoxicity effectors, and antiviral gene expression. Further studies should focus not only on the fish immune response but also on the pathogen strain, going deeper into the pathogenesis and depicting the pathogen or fish determinants for susceptibility/resistance.

4. Vaccination

One of the key aspects for the success of aquaculture is the design and generation of preventive vaccines to be commercially available and applied in fish culture. This Special Issue features one study focusing on vaccines, in which Strem et al. [16] designed vaccines for *Vibrio harveyi* and evaluated the effectiveness in the flathead grey mullet (*Mugil cephalus*), a relevant cultured fish suffering outbreaks and mortality caused by this pathogen. They found that vaccination with heat-killed bacteria, with adjuvant, is more effective (100% protection) than vaccination with external or internal protein extracts. In addition, the sera antibody levels correlated quite well with the protection.

In conclusion, this Special Issue “Fish Immunology 3.0” provides relevant information about fish immunity in general and against pathogens in particular, and the data and tools generated will be of significant use and application for researchers as well as fish farmers.
